# An Inverse Correlation of Serum Fibroblast Growth Factor 19 with Abdominal Pain and Inflammatory Markers in Patients with Ulcerative Colitis

**DOI:** 10.1155/2020/2389312

**Published:** 2020-05-29

**Authors:** Magdalena Panek-Jeziorna, Agata Mulak

**Affiliations:** Department of Gastroenterology and Hepatology, Wroclaw Medical University, Wroclaw, Poland

## Abstract

**Background and Aims:**

Bile acids (BA) play an important role in the modulation of numerous gut functions. Fibroblast growth factor 19 (FGF19) is the ileal hormone regulating BA homeostasis. The aim of the study was to evaluate serum FGF19 level and its correlation with clinical and endoscopic disease activity indices along with inflammatory biomarkers including serum CRP and fecal calprotectin levels in patients with ulcerative colitis (UC).

**Methods:**

Fasting serum FGF19 level was measured using ELISA test in 16 patients with active UC (7 F, 9 M), 15 patients with nonactive UC (8 F, 7 M), and 19 healthy controls (11 F, 8 M). The disease activity was assessed based on the clinical and endoscopic evaluations as well as serum CRP and fecal calprotectin level measurement.

**Results:**

The median serum FGF19 level was higher in patients with nonactive UC (175.3 pg/ml (108.7-342.3)) than in patients with active UC (114.3 pg/ml (68.9-155.3), *p* = 0.093). The median FGF19 level in healthy controls amounted to 151.6 pg/ml (90.6-224.2), and there were no statistically significant differences between the patients with active and nonactive UC compared to the healthy controls. An inverse correlation was observed between FGF19 level and abdominal pain intensity (*R* = –0.48, *p* = 0.007) as well as fecal calprotectin (*R* = –0.38, *p* = 0.036) and CRP levels (*R* = –0.36, *p* = 0.045). The serum FGF19 level was not correlated neither with clinical nor endoscopic disease activity indices.

**Conclusions:**

The inverse correlations between FGF19 level and abdominal pain as well as inflammatory markers in UC may imply its potential analgesic and anti-inflammatory effects.

## 1. Introduction

The results of recent studies have shed new light on the role of bile acids (BA) in the regulation of numerous gut functions including gastrointestinal motility, visceral sensitivity, secretion, inflammatory response, and gut barrier integrity [1–3]. Complex interactions between BAs and the gut microbiota participating in their transformation play also an important role [3–6]. BA malabsorption occurs in approximately 30% of patients with chronic diarrhea [7, 8]. Among patients with inflammatory bowel diseases (IBD), up to now, the role of BA malabsorption has been proved in the pathogenesis of diarrhea in patients with Crohn's disease, particularly after resection of the ileum [9]. The overload of nonabsorbed BAs entering the colon lumen induces water and electrolyte secretion, stimulating also colonic contractility. Some scarce data on BA malabsorption in ulcerative colitis (UC) remain ambiguous [9–11]. The role of BAs in the pathogenesis of other than diarrhea symptoms in IBD is unclear too.

A better understanding of the regulatory mechanisms in BA synthesis and enterohepatic circulation has enabled to introduce a new test for diagnosis of their malabsorption, which is the evaluation of serum fibroblast growth factor 19 (FGF19) concentration [12–14]. FGF19 is released from the epithelial cells of the ileum in response to the farnesoid X receptor (FXR) activation by absorbed BAs. In case of BA malabsorption, serum FGF19 level decreases, which results in increased BA synthesis in the liver [8, 15, 16]. It may additionally exacerbate bowel symptoms due to increased BA concentration in the colon. Furthermore, it has been shown that inflammation inhibits the FXR activation, while FXR agonists exert anti-inflammatory effect [3]. Therefore, disturbances within the gut-liver axis and FXR-FGF19 interaction may have significant diagnostic and therapeutic implications in IBD.

The evaluation of IBD activity includes the assessment of inflammatory markers as well as clinical features such as the intensity of diarrhea and abdominal pain. The gut immune system activation is directly associated with disturbances in intestinal barrier integrity and induction of visceral hypersensitivity. Potentially, the abovementioned anti-inflammatory effect of FXR activation resulting in FGF19 level increase [3] may contribute to the modulation of visceral pain response.

The main aim of the study was to evaluate fluctuation of BA concentration in active and nonactive phase of UC using serum FGF19 level measurement. Correlations between serum FGF19 level and main UC symptoms, clinical and endoscopic activity indices, and laboratory markers of inflammation such as fecal calprotectin and serum CRP levels were also assessed.

## 2. Materials and Methods

### 2.1. Subjects

Thirty-one patients with UC hospitalized at the Department of Gastroenterology and Hepatology at Wroclaw Medical University (Poland) and 19 healthy controls (11 F, 8 M; mean age 39) were recruited in the study. The UC patients were divided into 2 subgroups: 16 patients with active UC (7 F, 9 M; mean age 38) and 15 patients with nonactive UC (8 F, 7 M; mean age 46). The study was approved by the local Ethics Committee (KB-682/2015). A written informed consent was obtained from all participants prior to the study enrollment.

All subjects provided stool and fasting blood samples. The disease activity was assessed based on the clinical and endoscopic evaluations using the Rachmilewitz index and the Mayo Endoscopic Score, respectively. The predominant stool type and mean level of abdominal pain intensity over the last 7 days before examination were evaluated using the Bristol Stool Form Scale and the Visual Analog Scale (VAS), respectively. The prevalence of gastrointestinal symptoms, concomitant disorders, and medications in UC patients was assessed based on a questionnaire. The following features were considered the exclusion criteria: primary sclerosing cholangitis, ileal resection, and other severe conditions that could affect BA metabolism and circulation.

### 2.2. Quantitative Evaluation of FGF19 and Fecal Calprotectin

The quantitative evaluation of serum FGF19 and fecal calprotectin was performed by immunoenzymatic methods: Human FGF-19 ELISA (BioVendor, Laboratorni medicina a.s., Czech Republic) and EK-CAL (Bühlmann Laboratories, Switzerland), respectively. The patients were divided into active and nonactive subgroups based on the cutoff value of 250 *μ*g/ml for fecal calprotectin.

### 2.3. Statistical Analysis

Nonparametric statistics were used, and results are expressed as median along with the lower and upper quartiles (25Q-75Q). The Mann-Whitney *U* test was applied to compare differences in serum FGF19 and inflammatory markers between the groups. For the comparison of differences in frequency of abnormal results between the groups, the chi-squared test was used. The Spearman rank correlation coefficient (*R*) was also calculated to test associations between variables.

## 3. Results

The main characteristics regarding bowel symptoms in UC patients are presented in Table 1. The median VAS scores for abdominal pain during 7 days preceding the examination amounted to 0 (0-4) in patients with nonactive UC vs. 4.5 (2-6.5) in patients with active UC (*p* = 0.028).

The mean score according to the Rachmilewitz index used for the disease activity evaluation amounted to 1.3 ± 1.5 (median = 1) in nonactive UC and 7.6 ± 2.7 (median = 7) in active UC. Based on endoscopic assessment of the disease activity using the Mayo Endoscopic Score, only in 40% of patients with nonactive UC endoscopic remission was found (0 points). In 40% of subjects with nonactive UC, the Mayo Score amounted to 1, and in 20% to 2 points. In patients with active UC, the Mayo Score amounted to 2 in 37.5% of subjects and to 3 points in 62.5%. The majority of subjects with active UC (75%) had pancolitis, but without backwash ileitis.

**Figure 1:**
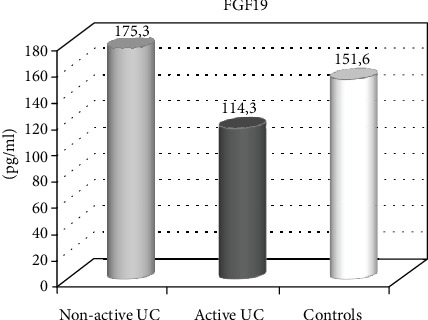
Median FGF19 level in the subgroups of UC patients and the controls. The median serum FGF19 level was lower in patients with active UC than in patients with nonactive UC (*p* = 0.093). There were no statistically significant differences between the patients with active (*n* = 16) and nonactive UC (*n* = 15) compared to the controls (*n* = 19).

Analyzing the serum FGF19 level in UC patients, a clear tendency was revealed that the median FGF19 level was lower in active UC (114.3 pg/ml) than in nonactive UC (175.3 pg/ml) (*p* = 0.093). The median FGF19 level in the healthy controls amounted to 151.6 pg/ml, but there were no statistically significant differences between the patients with active and nonactive UC compared to the controls (Figure 1). Despite the fluctuation of the FGF19 level depending on the disease activity, in the majority of UC patients, it was still within the normal range. An increased FGF19 level was found in 3 patients with nonactive UC, while a decreased FGF19 level was demonstrated in one patient with active UC and one patient with nonactive UC.

The median serum CRP and fecal calprotectin levels were significantly higher in active UC compared to nonactive UC (19.4 vs. 2.2 mg/l and 1974.3 vs. 87.1 *μ*g/g, respectively; *p* < 0.001).

**Figure 2:**
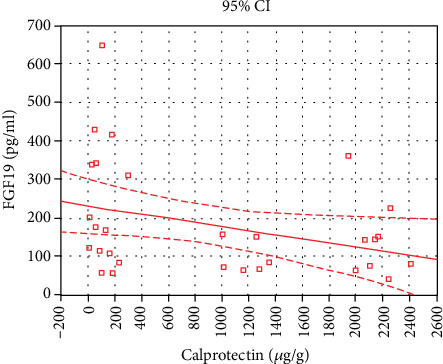
Correlation between serum FGF19 and fecal calprotectin levels in ulcerative colitis. The inverse correlation between serum FGF19 and fecal calprotectin levels was observed in UC patients (*n* = 31) (*R* = –0.38, *p* = 0.036).

**Figure 3:**
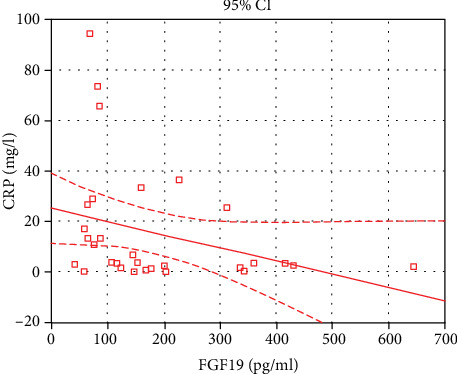
Correlation between serum FGF19 and serum CRP levels in ulcerative colitis. The inverse correlation between serum FGF19 and serum CRP levels was observed in UC patients (*n* = 31) (*R* = –0.36, *p* = 0.045).

An important part of the analysis was the evaluation of correlations between the serum FGF19 level as a new marker of disturbances in BA absorption and (1) stool frequency, (2) stool consistency based on the Bristol Stool Form Scale score, (3) abdominal pain, (4) clinical and endoscopic activity of the disease, and (5) calprotectin and CRP levels. The correlation analysis was performed combining the subgroups of patients with nonactive UC and active UC (total *n* = 31). The serum FGF19 level was not correlated neither with number of stools per 24 hours (*R* = –0.24; *p* = 0.189), the Bristol Stool Form Scale score (*R* = –0.26; *p* = 0.154), the Rachmilewitz disease activity index (*R* = –0.33; *p* = 0.073), nor with the Mayo Endoscopic Score (*R* = –0.28; *p* = 0.126). An inverse correlation in UC patients was found between the serum FGF19 level and abdominal pain intensity (*R* = –0.48, *p* = 0.007). Similarly, the inverse correlations were observed between serum FGF19 and fecal calprotectin (*R* = –0.38, *p* = 0.036) (Figure 2) and CRP levels (*R* = –0.36, *p* = 0.045) (Figure 3).

## 4. Discussion

The main finding of the study is that the serum FGF19 level in UC patients fluctuates depending on the disease activity with a clear tendency to be lower in active UC (114.3 pg/ml) than in nonactive UC (175.3 pg/ml) (*p* = 0.093). Despite this fluctuation in the majority of UC patients, the FGF19 level was still within the normal range and no statistically significant differences between any of the UC patient subgroup and the controls were revealed. Based on the available literature data, it has been estimated that BA malabsorption is present in about 1% of UC patients [17]. In two recent studies, it has been shown that the FGF19 level was normal [17] or slightly elevated [18] compared to the controls which is consistent with our own preliminary results [19]. In the current study, primary sclerosing cholangitis was an exclusion criterion and in none of the patients with nonactive UC, any signs of cholestasis were detected. Nevertheless, it cannot be totally ruled out that the increased FGF19 level found in 3 subjects with nonactive UC could be a prodromal sign of the biliary tract pathology. In the physiological conditions, FGF19 is mainly released by the ileum; however, in cholestasis, this hormone is also produced in the liver [20].

The available data on the role of BA in the pathogenesis of UC are not fully consistent that partially may result from the heterogeneity of the patient groups, small sample size, and some methodological differences [11]. In an old study published in 1971, Miettinen [21] postulated that diarrhea in UC is not associated with the loss of BAs in feces, but rather with colonic mucosa injury resulting in disturbances in absorption and increased fluid production to the gut lumen. At the same time, the author claimed that BA malabsorption is limited only to the subgroups of UC patients with backwash ileitis and after proctocolectomy with ileal pouch due to shorter gastrointestinal transit time and significantly smaller absorption surface [21]. In another study conducted in patients after ileorectal anastomosis, alterations in fecal BA profile characterized by decreased level of secondary BAs have been detected [22]. In physiological conditions, secondary BAs are produced by the colonic microbiota. Noteworthy, a growing body of evidence confirms a key role of the gut microbiota in BA metabolism in the gut lumen [23]. In a mouse model, it has been shown that the gut microbiota modulation induced by the administration of probiotics (VSL#3) enhanced BA deconjugation and fecal excretion [23]. These effects were associated with increased hepatic BA neosynthesis resulting from repression of the FXR-FGF15 axis (FGF15 is the murine homolog of FGF19), and treatment with a FXR agonist normalized fecal BA levels in probiotic-administered mice [23]. Of note, only conjugated BAs can be actively absorbed in the ileum, while in the colon, passive transport of secondary BAs occurs [20].

The results of studies in which alterations of BA levels in the serum in UC patients were investigated are also not convergent. Gnewuch et al. [10] performing liquid chromatography in 161 UC patients did not find significant differences in serum BA profile compared to the controls, except for decreased total BA tauroconjugate and unconjugated BA levels, which constitute only a small percentage of the serum BA pool [10]. In two other studies in UC patients, increased serum primary BA level [24] and decreased total serum BA level [25] were reported. However, Gothe et al. [26] assessing BA malabsorption by 7 *α*-hydroxy-4-cholesten-3-one (C4) did not reveal any significant difference between pediatric IBD patients compared to the controls.

Based on the evaluation of the colonic mucosa biopsies in UC patients with active pancolitis, downregulation in mRNA expression for the main ileal BA transporter—the apical sodium-dependent BA transporter (ASBT)—was found together with decreased activity of BA-detoxifying enzymes [27]. Such changes were not observed in nonactive UC or left-sided UC. Simultaneously, no changes in FXR expression were reported [27]. Moreover, Nijmeijer et al. [28] did not find any changes in FXR expression, but they observed alterations in FXR activation. The decreased FXR activation may impair FGF19 production that was observed also in the current study.

The data on the direct influence of BAs on the clinical course of different forms and phases of IBD remain scarce. Therefore, one of the main aims of this study was to analyze the correlation between the serum FGF19 level and main UC symptoms including diarrhea and abdominal pain, clinical and endoscopic disease activity, and inflammatory markers. The serum FGF19 level was not correlated neither with number of stools per 24 hours nor with the Bristol Stool Form Scale score. To the best of our knowledge, this is the first report on the negative correlation between the FGF19 level and abdominal pain intensity. Previously, it has been shown that activation of TGR5—a membrane-type receptor for BAs—mediates BA-induced itch and analgesia [29]. Relatively higher FGF19 level in patients with nonactive UC, despite the presence of endoscopic signs of colonic mucosa inflammation in 60% of them, could point to the potential analgesic effects of FGF19.

Analyzing the correlation of the FGF19 level with the Rachmilewitz disease activity index, some trend was observed, but without statistical significance (*R* = –0.33; *p* = 0.073). Gothe et al. [26] did not reveal any correlation between C4 level as a marker of BA malabsorption and clinical IBD activity neither; however, their study was conducted in children with the use of different scales to score the disease activity. Furthermore, in our study, no correlation was found between the FGF19 level and the Mayo Endoscopic Score (*R* = –0.28; *p* = 0.126), which has not been evaluated so far.

One of the most interesting findings of the current study in UC patients is the negative correlation between FGF19 and inflammatory markers levels including fecal calprotectin (*p* = 0.036) and serum CRP (*p* = 0.045). The lower FGF19 level in patients with active UC (although in the majority of subjects still within the normal range) could be associated with decreased BA absorption resulting in increased BA pool in feces. In the colon, the gut bacteria participate in the secondary BA production. Interestingly, antibacterial properties of BAs depend on their profile in the fecal pool, whereas dysbiosis present in IBD may contribute to alterations of BA transformation [6]. Moreover, BAs as ligands for transcription factors modulate the expression of genes involved in BA transformation including FXR, which may exert a direct immunomodulatory effect. On the other hand, proinflammatory cytokines may repress FXR expression inducing disturbances in BA absorption [30], which suggests a complex causative relation between BA malabsorption and gut inflammation intensity. Gothe et al. [26] did not reveal any correlation between the C4 level and inflammatory markers in UC. In this study, for the first time, the correlation between FGF19 and fecal calprotectin levels was evaluated and a negative correlation between investigated parameters has been found.

Potentially, a higher FGF19 level in nonactive UC could be associated with stimulation of its excretion by steroidotherapy used to induce remission. In a rat model of IBD, steroid-dependent induction of ASBT expression has been shown [31]. Furthermore, it has been demonstrated that in healthy volunteers, 21-day treatment with budesonide induces an increase in ASBT expression (by 34%) in the ileum resulting in increased FGF19 production [32]. The increased FGF19 release in UC remission may exert anti-inflammatory effect as well as reduce BA synthesis in the liver and consequently BA concentration in the colon, which may alleviate the symptoms.

Noteworthy, BAs may induce a dual effect—induction or inhibition of inflammation [3]. The effect of BA action is determined by multiple factors such as concentration of BAs, their physicochemical properties, and interactions with the gut microbiota [2]. In a mouse model of UC, it has been demonstrated that experimental colitis may disturb BA synthesis by the negative feedback signaling within the FXR-FGF19 axis [33]. Recent findings have confirmed a crucial role of FXR in the modulation of inflammatory response and intestinal barrier integrity [34]. The results of both *in vivo* and *in vitro* studies have demonstrated that on the one hand, inflammation reduces FXR expression, while on the other hand, the activation of FXR exerts anti-inflammatory effect by reducing the production of proinflammatory cytokines [35]. Additionally, TGR5 membrane receptors present on enterocytes, enteric neurons, and immune cells also participate in the regulation of numerous gut functions. Therefore, anti-inflammatory effect induced by FXR and FGR5 agonists may be of clinical significance [3].

The fluctuation of the FGF19 level shown in the current study reflects changes in serum and fecal BA concentration. Importantly, fecal secondary BAs due to their cytotoxic effect are considered a risk factor for colorectal cancer, also in the course of IBD. Moreover, chronically increased FGF19 level has also been reported to increase the risk for both colorectal cancer and cholangiocarcinoma in IBD patients which may have relevant clinical implication [36, 37].

Among limitations of the study are relatively limited sample size and the fact that the subgroups of UC patients with active and nonactive phase of the disease constituted disjoint sets. However, the subgroups were very carefully characterized with respect to clinical and endoscopic disease activity and lab test results that enabled evaluation of numerous correlations between investigated features and parameters. The novelty of the study is related to the pioneer reports on the negative correlations between the FGF19 level and abdominal pain intensity as well as fecal calprotectin. The evaluation of FGF19 is a useful test to detect disturbances in BA absorption and circulation. The test is easy to perform and noninvasive, but a fasting blood sample is required due to postprandial increase in the FGF19 level [38].

## 5. Conclusions

The serum FGF19 level shows fluctuation depending on the disease activity, which indicates the association between the regulatory mechanisms of BA enterohepatic circulation and UC activity. The inverse correlations between the FGF19 level and abdominal pain as well as inflammatory markers may imply its potential analgesic and anti-inflammatory effects—direct or due to the FXR-FGF19 axis activation. The dynamic of the FGF19 level fluctuation depending on the UC phase suggests new therapeutic aims associated with the activation of FXR, which constitutes a key element of the gut-liver axis.

## Figures and Tables

**Table 1:** Bowel symptoms in patients with ulcerative colitis (UC).

Symptoms	Number of patients reporting symptoms (%)		*p*-value
	Nonactive UC *n* = 15	Active UC *n* = 16	Active UC vs. nonactive UC
Number of stools^∗^			
Median	3	9	0.00003
Max	8	20	
Bristol Stool Form Scale^∗∗^ (median)	4	7	0.00122
Blood in stool^∗∗^	4 (27%)	14 (88%)	0.0006
Mucus in stool^∗∗^	4 (27%)	12 (75%)	0.00712
Abdominal pain^∗∗^	5 (33%)	10 (63%)	0.104
Abdominal discomfort^∗∗^	8 (53%)	12 (75%)	0.208
Bloating^∗∗^	4 (27%)	6 (38%)	0.519

^∗^Mann-Whitney *U* test, ^∗∗^*χ*^2^ test.

## Data Availability

The data used to support the findings of this study are included within the article. Additional data are available from the corresponding author (agata.mulak@wp.pl).
